# Psychosocial Profile in Portuguese Adolescents with Chronic Disease Attending an Outpatient Department in a Hospital Setting

**DOI:** 10.1155/2018/9382648

**Published:** 2018-12-16

**Authors:** Teresa Santos, Margarida Gaspar de Matos, Adilson Marques, Celeste Simões, Isabel Leal, Maria do Céu Machado

**Affiliations:** ^1^FMH, Faculdade de Motricidade Humana (Equipa Aventura Social)/Universidade de Lisboa, Lisboa, Portugal; ^2^William James Center for Research, ISPA-Instituto Universitário, Lisboa, Portugal; ^3^ISAMB, Instituto de Saúde Ambiental, Faculdade de Medicina, Universidade de Lisboa, Lisboa, Portugal; ^4^INSERM/University Paul Sabatier (Sabbatical Grant Foundation for Science and Technology (FCT-SFRH/BSAB/135160/2017)3, Toulouse, France; ^5^Faculdade de Medicina da Universidade de Lisboa, Departamento de Pediatria do Hospital de Santa Maria, CAML, Centro Académico de Medicina de Lisboa, Lisboa, Portugal

## Abstract

Living with a chronic disease (CD) in adolescence involves new multifaceted challenges. This study aims to conduct a psychosocial characterization of a group of adolescents with chronic diseases in a hospital setting and to compare such dimensions for the total group and for different diseases. A cross-sectional study included 135 adolescents with chronic diseases (51.9% boys; 48.1% girls), having an average age of 14±1.5 years (SD=1.5) and attending a paediatric outpatient department in a hospital setting. Statistically significant differences were found among the different chronic diseases for the variables self-regulation (adolescents with diabetes had significantly higher competencies) and multiple psychosomatic symptoms (adolescents with neurologic diseases reported significantly more complaints). Boys presented both better health-related quality of life and psychosomatic health when compared to girls. No statistically significant differences were observed for health-related quality of life, psychosomatic health, resilience, and social support. These findings bring important suggestions especially while planning interventions, which must take into account the promotion of a healthy psychosocial development, through an inclusive perspective (covering different chronic diseases), that take into consideration specific and gendered approaches. Such suggestions might help healthcare professionals to better plan interventions in order to increase their effectiveness.

## 1. Introduction

Mortality rates of several fatal chronic diseases have decreased over the past 40 years due to advances in paediatric medicine and in life expectancy [1]. Around 10% of adolescents suffer from a chronic disease affecting daily life, and this prevalence will likely tend to further increase [2], assuming a global public health concern [3]. Living with a chronic condition in adolescence, where profound bio-psycho-social changes occur, involves a whole set of new multifaceted challenges to youth, such as learning daily routines and functioning, adopting healthy life styles, monitoring symptoms [4], and dealing with uncertainty about the disease itself and the future. It is a demanding experience for adolescents, which can affect several aspects of the individual's life, and the adjustment on multiple domains, as well as the regulation of cognitions, emotions, behaviours, physiologic aspects, and interaction with others [5]. Therefore, a chronic condition in adolescence may compromise a healthy psychological development in adolescents [6, 7], which can experience more adjustment difficulties [8], a poor health-related quality of life (HRQoL) [9, 10], and a negative impact on the general quality of life (QoL) [11]. Moreover, the diagnosis, treatment, and ongoing management of chronic diseases are stressful for youth, families, and healthcare professionals [3, 12, 13]. Thus, this group is doubly disadvantaged and may be more vulnerable to adverse health outcomes [14, 15]. Such vulnerability is observed in previous Portuguese studies [16–18], more specifically, in the area of chronic condition [19, 20].

The impact of living with a chronic disease can bring losses to adolescents' quality of life. Literature suggests that studies focusing on children's subjective well-being should include the associations among demographic variables (e.g., gender, age, and socioeconomic status), interpersonal characteristics (e.g., self-perceptions, psychological well-being, mood, and emotions), and the perception of well-being and happiness [21, 22]. Furthermore, the individual perception regarding the disease and its adaptation process can be changeable and moderated by gender [23]. Girls can be at a higher risk for a more compromised psychological adjustment, reporting low life satisfaction, multiple health complaints, and poorer outcomes for self-rated health and health-related quality of life, when compared with boys [24–26]. This may be explained by different internalization and externalization patterns, as well as gender-specific experiences on puberty [27, 28]. Additionally, resilience can also play an important role in the adaptation to adverse health states, leading to a more acceptable quality of life [29, 30]. 

The research comparing adolescents with and without a chronic condition, or among different conditions, has been contradictory, inconclusive, and not confirming a direct relationship between the degree of suffering and the chronic health condition [31]. Responses are quite variable and not homogeneous, depending on various specific individual/contextual factors and on the type of condition and emerging limitations, being even worse in the presence of both [32]. In addition, the prevalence of different health conditions, namely, having a greater number of concurrent health problems (3/more compared with 2), is associated with worse quality of life, as well as with the type of chronic condition [33, 34]. Particularly, the results on emotional well-being and psychosocial development of these adolescents are still controversial and variable, due to the definition of chronic disease [35] and its state of complexity, and also because of the numerous instruments used to assess psychological well-being [36]. In addition, reports generally focus on a single set of conditions, or on very large populations [35]. Although some evidence highlights the increased vulnerability in groups of adolescents with chronic disease, other results suggest that they can be better than healthy individuals and presenting a good psychosocial functioning [36, 37].

Psychosocial factors increase their importance compared to physical influences as individuals age. Therefore, in the promotion of adolescents' health, a major focus was underlined on positive guidelines identifying important areas such as the perception of subjective well-being and social support (which affect HRQoL) [38, 39]. Health professionals have increasingly recognized and acknowledged psychosocial variables, rather than being mainly focused on physical dimensions [31, 40–43]. This recognition occurred because theoretical models have shown that an association of multiple psychosocial factors can have a higher impact on adult's health [18, 44]. Thus,* psychosocial* is the shorthand term for the combination of psychological and social dimensions [45], which has been widely used in literature related to health outcomes (Kojima et al., 2009), ultimately based on the World Health Organization's (WHO) definition of health, as a state of complete physical, mental, and social well-being, and not merely the absence of disease and infirmity [46].

Taking into account the increasing relevance of psychosocial domains, the present study focuses on such variables, including health-related quality of life, individual symptoms of physical/psychological functioning (psychosomatic complaints), resilience, self-regulation, and social support. Specifically, the aims of this study were (1) to make a psychosocial characterization of a group of adolescents with chronic diseases and (2) to compare the study's variables within the total group of adolescents, and in the three chronic disease's subgroups, divided according to their medical condition (asthma, diabetes, and neurologic diseases). The focus on these disease groups relies on the literature's evidence as the most prevalent chronic diseases in adolescence [32].

## 2. Methods

### 2.1. Participants, Design, and Procedure

This cross-sectional study included 135 selected chronic adolescent patients (51.9% boys; 48.1% girls) with a mean age of 14 years (*SD*=1.5), attending a clinical appointment in the paediatric outpatient department of* Centro Hospitalar Lisboa Norte* – CHLN EPE (North Lisbon Central Hospital). The majority were boys (51.9%;* n*=70), had normal weight (71.8%;* n*=94), were of Portuguese nationality (97.8%;* n*=132), lived in Lisbon (84.4%;* n*=114), and attended the 7^th^-9^th^ school grades (53.3%;* n*=72). Health professionals (paediatricians) help to identify the following applied inclusion criteria for this study: (1) diagnosis of chronic disease (included in the specific medical appointments of diabetes, allergy, and neurology); (2) age between 12 and 16 years at the time of the study; (3) having cognitive skills to fill the questionnaire autonomously. Consequently, adolescents and their parents who met the inclusion criteria were approached directly by their health professionals (physicians and/or nurses) during appointment, in order to participate in the study.

The participation was voluntary, the description of the study's aims and participants' rights was delivered, and the informed consent required by the ethical committee was filled, by both adolescents and their parents. Data was collected using a self-report questionnaire after or before the medical appointment, according to the most convenient moment for all (adolescent, parents, and health professional's appointment), whenever possible in an individual and more quiet medical office. Ethical approval for this study was obtained from The Ethics Committee for Health from CHLN-EPE, the institution's ethical committees (Compliance with Ethical Standards: Reference PCA-12 Nov.2012-0785).

### 2.2. Measures

Adolescents completed self-report questionnaires to assess sociodemographic, clinical, and psychosocial variables. Sociodemographic and clinical variables included age, gender, geographic region, nationality, educational level (adolescents and parents), time since diagnosis, and the use of special equipment and/or medication related to the chronic condition. The psychosocial variables included the Health-related quality of life-HRQoL (KIDSCREEN-10 Index) [16, 47, 48], Psychosomatic Complaints (Scale Symptoms Check List-HBSC-SCL), Symptoms Check List (SCL-HBSC) [49–51], Resilience (Scale Healthy Kids Resilience Assessment Module) [52, 53], Self-regulation (Scale Adolescent Self-Regulatory Inventory-ASRI) [54], and Social Support (Scale of Satisfaction with Social Support-SSSS) [38, 55]. The Scale Symptoms Check List (SCL-HBSC) was used in the international study HBSC/WHO [56, 57]. These instruments are presented in more detail in Table 1.

### 2.3. Statistical Analysis

Descriptive statistics (means, standard deviation and percentage) were used to characterize the sample. Adolescents were classified by medical conditions into three groups: diabetes, allergy, and neurologic diseases. All data was tested for normality prior to any analyses. The normality of the variables was tested by Shapiro-Wilk and Kolmogorov-Smirnov tests, and variance homogeneity was tested by Levene's test. ANOVA (followed by comparisons using the Bonferroni Post Hoc Test) and Student* t*-test were performed to evaluate significant differences in the analysed variables among the three groups of diseases and for the total group. Kruskal-Wallis tests were additionally conducted when normality and the variance homogeneity of the variables were not observed. Data analysis was performed using the Statistical Package for Social Sciences (SPSS), version 22.0, for Windows. For all tests statistical significance was set at* p*<0.05.

## 3. Results

### 3.1. Sociodemographic and Clinical Variables

The sociodemographic and the clinical characteristics of the sample are presented in Table 2.

The sample included a group of adolescents divided into three subgroups of chronic diseases: diabetes (31.9%;* n*=43), allergic diseases (46.7%;* n*=63), and neurologic diseases (21.5%;* n*=29). The total group had a mean time of diagnosis of 7.5 years (*SD*=4.7), with a majority of patients who take medication (65.2%;* n*=88) and generally did not use special equipment (61.5%;* n*=83) related to the chronic disease. The group of adolescents with allergic diseases reported the higher percentage of taking medication due to the disease (96.8%;* n*=61). The adolescents with diabetes showed the higher percentage of using special equipment due to the disease (97.7%;* n*=42) (e.g., tolls related to the monitoring of blood glucose and the intake of insulin).

### 3.2. Psychosocial Variables

The group comparisons (Table 3) showed significant statistical differences for the variables Self-regulation (Scale Adolescent Self-Regulatory Inventory-ASRI) and the group of adolescents with diabetes had a significantly higher level of self-regulation for the total score [*F*(2,132 = 3.598,* p*=.030)], as for the short-term regulation score [*F*(2,132; 4.091,* p*=.019)]. Post hoc tests indicated that, compared with the group of adolescents with allergic diseases, the adolescents with diabetes had significantly higher values for both the total score (124.7 ± 15.6* vs. *117.5 ± 14.7) and short-term dimension (44.2 ± 7.0* vs.* 40.5 ± 6.7) of self-regulation competences; and no significant differences were found for the group of neurologic diseases. Also for the variable SCL-MC (two or more symptoms, more than once a week in the past six months) there were statistically significant differences between the different groups of diseases, *χ*^2^(2)=6.095,* p*=0.047.

No statistically significant differences were found in the three subgroups of chronic diseases concerning the psychosocial variables: Health-related quality of life-HRQoL (KIDSCREEN-10 Index), Psychosomatic Complaints (Scale Symptoms Check List–HBSC-SCL), Resilience (Scale Healthy Kids Resilience Assessment Module), and Social Support (Scale of Satisfaction with Social Support-SSSS).

Facing the few statistic differences observed in psychosocial variables, further analyses considering the total group of adolescents were conducted for the sociodemographic and clinical variables. Results are presented in Table 4. Boys had statistically significantly higher health-related quality of life than girls (82.43 ± 11.15* vs*. 76.68 ± 13.21;* t*(133)=2.740,* p*=0.007). Boys also presented a statistically significantly better health (reporting less psychosomatic complaints), when compared to girls (36.57±4.52* vs*. 34.51 ± 4.99;* t*(133)=2.523,* p*=0.013). Concerning the use of special equipment related to the chronic disease, the group of adolescents who uses special equipment had statistically significantly higher self-regulation competences when compared to the group of adolescents who does not use special equipment (123.31 ± 15.03* vs*. 117.77 ± 13.66;* t*(133)=-2.210,* p*=0.029).

## 4. Discussion

Taking into account the present results, for the three chronic disease groups, significant differences were found for the variables SR and SCL-MC. Regarding SR, the group of adolescents with diabetes showed significantly higher levels of self-regulation, both for the total score and for the short-term dimension. In this group it is also observed that a higher percentage of adolescents use disease-related special equipment (e.g., insulin assessment/intake tools). Previous literature can help to explain the higher level of self-regulation, once it is suggested that adolescents with diabetes often have to adhere to multiple complex daily tasks, including blood glucose monitoring, insulin administration, nutrition management, and efforts to practice physical activity. All of these demands require management skills and high levels of self-regulation, a variable actually considered as a fundamental component of diabetes care, with an important association with better metabolic control in youth [58]. Concerning SCL-MC, the results indicated that the group of adolescents with neurologic diseases reported higher multiple psychosomatic health complaints, reinforcing prior studies pointing out that having a greater number of health problems (3/more compared with 2) [33, 34], for example, the type of disease and its emerging limitations [32], can lead to worse responses. Additionally, neurological diseases are probably the ones that present higher visible limitations, compared to others, and this fact has been shown in literature, to primarily determine the adolescent's perception of the diseases' severity [36].

No statistically significant differences were found for the three chronic disease groups, concerning the psychosocial variables KIDS-10, SCL, RES, and SSSS. These findings are in accordance with prior research with adolescents with chronic diseases that reported no significant associations [59, 60] or when comparing different diseases showed a reasonable psychosocial functioning, a general well-adjustment, and a less severe self-perception of the disease as physicians did [36], although other studies have indicated a high risk of impairment in psychosocial functioning for adolescents with chronic conditions [61–63]. Thus, it reinforces the idea that the activity of the disease may not be associated with psychosocial factors and fails to reveal the perceived physical and mental quality of life of adolescents [64]. At the same time, it emphasizes previous research not confirming a direct relationship between the degree of suffering and the condition (in comparison of adolescents with different conditions) [31, 35, 36]. In practice, it may be difficult for physicians to accurately infer the patient's perception of the disease's severity [36].

With respect to the total group of adolescents included in this study, relatively moderate to high levels of health-related quality of life and social support were found, in accordance with previous studies with adolescents with chronic conditions [37, 65, 66], and in a similar tendency of the national healthy population, when observing the Portuguese data [47]. Such results may be due to the fact that, when assessing the quality of life in adolescents with chronic diseases, some aspects of the disease and its limitations are sometimes underestimated. Indeed, disease modifies adolescents' experiences due to treatments and limitations and also influences the subjective perception of life, social relationships, goals, priorities, and self-esteem, being one major stressful event, but it can, at the same time, promote personal maturity. Literature also suggests that developmental needs are the same for both healthy and chronically ill adolescents, probably implying the existence of a similar structure of quality of life in such population, once health, physical, and mental well-being are crucial dimensions [67]. 

Additionally the present results indicate gender differences for the total group of adolescents, being in accordance with previous research suggesting that coping with a chronic disease is a changeable process, moderated by gender [21–23]. Boys reported a significantly higher health-related quality of life and less psychosomatic complaints, compared with girls. Such evidence is in line with prior studies pointing out that girls can be at higher risk for a more compromised psychological adjustment, reporting poorer health/mental health outcomes, multiple health complaints, and more risk-internalizing behaviours [24–28]. Lastly, the group of adolescents who uses special equipment reported higher self-regulation competences than the ones who do not use special equipment. These results may be explained because the group who uses special equipment is mostly composed of adolescents with diabetes (97.7%).

### 4.1. Limitations and Strengths

This study has a number of limitations to be considered. The nonrepresentative sample and the cross-sectional design preclude inferences concerning causality, presenting weakness to examine the direction of the effects; therefore, plausible generalizations should take this into consideration. Longitudinal data would be needed. Finally, the group of adolescents was heterogeneous in terms of the type/severity of the disease, and findings were entirely based on adolescents' self-reports; thus, biases in perception and reporting cannot be ruled out. Regardless of these limitations, this study allows increasing the knowledge on clinical evidence and giving important preliminary insights on psychosocial dimensions in a group of adolescents with chronic conditions, in a hospital setting. It offers important suggestions to help healthcare professionals, especially while planning interventions. In the future, it would be important to test these variables in a larger sample and to conduct comparisons with other chronic conditions or with different degrees of disease's severity, as well as with healthy groups of adolescents. Larger comprehension on the role of gender is suggested because it seems to be a crucial explanatory variable, as also shown in previous research [37].

### 4.2. Conclusions

In conclusion, this study brings knowledge on the psychosocial impact of living with a chronic condition in a group of adolescents in a hospital setting and allowed the identification of the most vulnerable groups of adolescents considering the different diseases. It further reinforced that, more than deducing psychosocial impairment mainly from the disease's characteristics, it is important to frequently assess such dimensions [31, 40–43] by multidisciplinary teams, to help cope with the disease [18, 43, 44], regarding general health outcomes [64]. Such assessment should include the adolescent's self-perceptions [64], because health promotion implies effective and active support for physical, psychological, and social well-being of children and adolescents, matching their own needs, and, furthermore, due to variable and heterogeneous responses of adjustment to a chronic disease, which depend on various specific individual/contextual factors, and on the type of condition, onset of diagnosis, and emerging limitations [32].

It seems crucial to implement programs/interventions to promote a healthy psychosocial development and equal psychoeducation. Facing this study's results and the previous studies that suggest both differences/similarities on psychosocial impairment when comparing different chronic diseases, it may be useful to plan inclusive interventions that concurrently include individual and gendered aspects. To carry out such interventions on a more individualized assessment of each adolescent's psychosocial status could help to increase intervention's effectiveness, rather than mostly drawing it from the disease's activity. The present study emphasizes the need for self-regulation skills training, with a special attention on girls as the most vulnerable group. In a wider perspective, it can be suggested that healthcare professionals could design interventions which focus on minimizing difficulties and maximizing potential skills in adolescents with a chronic disease. For that purpose, it is crucial to include psychological and social dimensions, along with educational achievement [43].

## Figures and Tables

**Table 1:** Psychosocial variables.

**Name**	**Psychosocial Measure**	**Abbreviation (in this study)**	**Short Description**	**Alpha Cronbach**
KIDSCREEN-10 Index [16, 47, 48]	Health-related quality of life – HRQoL	KIDS-10	(i) Short version of KIDSCREEN-52; (ii) 10 items, on a 5-point Likert-type scale; (iii) Ranges from 0 to 100; (iv) Lower values reflect feelings of unhappiness, dissatisfaction and inadequacy. Higher values show feelings of happiness, perception of adequacy and satisfaction within adolescent's life contexts.	*α* = 0.83

Symptoms Check List (SCL-HBSC) [49–51]	Psychosomatic complaints (unidimensional latent trait).	SCL	(i) Used in the HBSC/WHO Study [56, 57] (ii) 8 items focusing on subjective physical and psychological health complaints; (iii) Each item answered on a 5-point Likert-type response scale; (iv) Resulting values between 1 (worst health) and 5 (best health); (v) Ranges from 8 to 40.	*α* = 0.78
Symptoms Check List (Multiple Complaints) [49]	Concerning the variable Psychosomatic Complaints (Scale Symptoms Check List – HBSC-SCL) and according to the literature that suggests that adolescents with recurrent multiple health complaints are considered to present noticeable subjective health complaints; an additional variable named SCL-MC (Multiple Complaints) was created, composed by those adolescents who reported two or more symptoms, more than once a week in the past six months.			

Healthy Kids Resilience Assessment Module [52, 53]	Resilience (2 dimensions: external and internal resources).	RES	(i) The present study only used the internal resources; (ii) 18 items answered on a 4-point scale; (iii) Ranges from 18 to 72; (iv) Higher scores indicate higher levels of competences, protection and resilience to adversity.	*α* = .0.72^*∗*^

Adolescent Self-Regulatory Inventory – ASRI [54]	Self-regulation (2 dimensions: Short term-SR-ST and Long term-SR-LT).	SR	(i) In this study the instrument was translated from the original English version into Portuguese language (and back translation). It was then revised by a group of specialized experts within the area and a pre-test in schools with a group of students was conducted. (ii) 36 items answered on a 5-point Likert scale; (iii) Ranges from 36 to 180. (iv) Higher values indicate better competences of self-regulation.	*α* = .0.79^*∗*^

Scale of Satisfaction with Social Support [38, 55]	Satisfaction with social support (2 dimensions: Satisfaction with Social Support-SSS; and Need for Activities connected to Social Support-NASS).	SSSS	(i) Translation and adaptation for children and adolescents, of a Satisfaction with Social Support Scale for adults; (ii) 12 items answered on a 5-point scale; (iii) Ranges from 18 to 72; (iv) Higher scores indicate higher satisfaction with social support (SSS) or higher satisfaction for not feeling the need to have more social support activities (NASS).	*α* = 0.85^*∗*^

*∗*: value for the total score of the scale.

**Table 2:** Participants' sociodemographic and clinical characteristics.

	**Subgroups of Chronic Diseases**			**Total Group of CC**
	**Diabetes**	**Allergic diseases**	**Neurologic Diseases**	
	N=43	N=63	N=29	N=135
***Sociodemographic Variables***				

**Age (years) (M±SD)**	13.7±1.6	14.2±1.5	14.0±1.5	14.0±1.5
**Gender (**%**)**				
Boy	41.9	58.7	51.7	51.9
Girl	58.1	41.3	48.3	48.1
**Educational Level – Adolescents (**%**)**				
Basic 2^nd^ Level (5^th^-6^th^ Grades)	23.3	20.6	20.7	21.5
Basic 3^rd^ Level (7^th^-9^th^ Grades)	48.8	58.7	48.3	53.3
Secondary Level (10^th^-12^th^ Grades)	27.9	20.6	31.0	25.2
**Educational Level - Father (**%**)**				
Basic Level (1^st^-9^th^ Grades)	55.0	69.5	67.9	64.6
Secondary Level (10^th^-12^th^ Grades)	27.5	23.7	17.9	23.6
Superior (or more) Level (University, Post-Graduate)	17.5	6.8	14.3	11.8
**Educational Level - Mother (**%**)**				
Basic Level (1^st^-9^th^ Grades)	46.3	62.9	44.8	53.8
Secondary Level (10^th^-12^th^ Grades)	34.1	24.2	34.5	29.5
Superior (or more) Level (University, Post-Graduate)	19.5	12.9	20.7	16.7

***Clinical Variables***				

**Time since diagnosis (years) (M±SD)**	5.0±3.9	8.9±4.6	8.5±4.9	7.5±4.7
**Special equipment (**%**)**				
No	2.3	93.7	79.3	61.5
Yes	97.7	6.3	20.7	38.5
**Medication (**%**)**				
No	79.1	3.2	37.9	34.8
Yes	20.9	96.8	62.1	65.2

**Table 3:** Differences between the groups of adolescents with diverse chronic diseases for all psychosocial variables.

	**Total**	**Subgroups of Chronic Diseases**			
		**Diabetes**	**Allergology**	**Neuropathology**	*p*
	N=135	N=43	N=63	N=29	
**KIDS-10 (M±SD)** ^**1**^	79.7±12.5	79.9±13.0	79.7±12.7	79.2±11.6	0.976
**SCL (M±SD)** ^**1**^	35.6±4.8	35.3±4.3	36.1±5.0	35.0±5.2	0,536
**SCL-MC (M±SD)** ^**2**^	0.67±1.18	0.60±1.2	0.54±1.1	1.07±1.3	0.047
**RES (M±SD)** ^**1**^	58.4±7.8	60.3±7.1	58.1±7.9	56.1±8.3	0.074
**SR (M±SD)** ^**1**^	120.0±14.4	124.7±15.6	117.5±14.7	118.2±9.8	0.030
SR-ST	41.8±6.8	44.2±7.0	40.5±6.7	41.1±6.0	0.019
SR-LT	50.2±7.7	51.6±8.1	49.6±8.0	49.5±6.2	0.345
**SSSS (M±SD)** ^**1**^	45.1±8.6	45.6±9.4	45.0±8.1	44.4±8.9	0.860
NASS	15.8±4.7	16.0±5.2	16.0±4.1	15.1±5.0	0.652
SSS	29.1±5.4	29.6±5.3	28.7±5.6	29.3±5.2	0.711

SCL: Symptoms Check List; SCL-MC: Symptoms Check List Multiple Complaints; KIDS-10: KIDSCREEN; RES: Resilience; SR: Self-regulation; SR-ST: Self-regulation, Short Term; SR-LT: Self-regulation, Long Term; SSSS: Scale of Satisfaction with Social Support; NASS: Need for Activities connected to social support; SSS: Satisfaction with social support.

^1^Tested by ANOVA followed by Bonferroni Post Hoc Test.

^2^Tested by Kruskal-Wallis.

**Table 4:** Differences between gender (boys/girls) and using special equipment (yes/no) for all psychosocial variables.

	**Gender**			**Special Equipment**		
**(M±SD)**	**Boys**	**Girls**	*p*	**No**	**Yes**	*p*
**KIDS-10** ^**1**^	82.43±11.15	76.68±13.21	.007	79.69±12.14	79.62±13.12	.974
**SCL** ^1^	36.57±4.52	34.51±4.99	.013	36.00±4.89	34.90±4.73	.202
**RES** ^1^	58.07±7.55	58.66±8.13	.663	57.70±7.64	59.40±8.04	.218
**SR** ^1^	119.63±15.17	120.18±13.64	.824	117.77±13.66	123.31±15.03	.029
**SSSS** ^**1**^	46.34±8.64	43.69±8.49	.075	44.61±7.86	45.80±9.80	.444

SCL: Symptoms Check List; KIDS-10: KIDSCREEN; RES: Resilience; SR: Self-regulation; SS: Social Support.

^1^Tested by independent *t*-test.

Note: no statistical differences were found for the variables: age, educational level of the adolescents, educational level of the mother, educational level of the father, time since diagnosis, and medication use related to disease.

## Data Availability

All data supporting this study was provided through an anonymous and confidential interview and questionnaire. Therefore, due to ethical concerns, supporting data cannot be made openly available. Further information can be required by contacting the authors.

## Abbreviations

CHLN EPE: Centro Hospitalar Lisboa Norte
HBSC/WHO: Health-Behaviour in School-aged Children/World Health Organization
HRQoL: Health-related quality of life
QoL: Quality of life
KIDS-10: KIDSCREEN Index
NASS: Need for Activities connected to Social Support
RES: Healthy Kids Resilience Assessment Module
SCL: Symptoms Check List-HBSC-SCL (Psychosomatic Complaints)
SR: Adolescent Self-Regulatory Inventory (ASRI)
SR-LT: Self-regulation, Long Term
SR-ST: Self-regulation, Short Term
SSS: Satisfaction with Social Support
SSSS: Scale of Satisfaction with Social Support
WHO: World Health Organization.
